# Accuracy of CT for measuring femoral neck anteversion in children with developmental dislocation of the hip verified using 3D printing technology

**DOI:** 10.1186/s13018-021-02400-x

**Published:** 2021-04-14

**Authors:** Zhencun Cai, Chengzhe Piao, Tianyu Zhang, Lianyong Li, Liangbi Xiang

**Affiliations:** 1 Department of Orthopaedics Surgery, General Hospital of Northern Theater Command, Shenyang, Liaoning Province China; 2 Department of Orthopaedics Surgery, Central Hospital of Shenyang Medical College, Shenyang, Liaoning Province China; 3 Department of Pediatric Orthopaedics, Shengjing Hospital of China Medical University, Shenyang, Liaoning Province China

**Keywords:** Femoral neck, Anteversion angle, 3D printing technology, CT measurement, Hip joint

## Abstract

**Background:**

Accurate femoral neck anteversion angle (FNA) measurement is of great significance in the diagnosis and treatment of developmental dysplasia of the hip (DDH) in children. The FNA measurement still remains a controversy. We aimed to verify the accuracy of our CT method by 3D printing technology and to evaluate its clinical value.

**Methods:**

Sixty-eight children with unilateral DDH were enrolled, and their FNA was measured using 2D-CT and 3D-CT, respectively, by three observers. This procedure was repeated 3 months later. The above measurement outcomes were then compared with the results in the 3D-printed femur (3D-PF) model. The FNA in the 3D-PF model was measured by three observers (two radiologists and one orthopedist; all were professors) collectively through electronic angle instrument.

**Results:**

The primary measurement of FNA at the affected hips by 2D-CT was 44.0 ± 6.1, 49.5 ± 8.9, and 52.8 ± 7.9°, respectively. On the 3D-CT, it was 47.6 ± 5.4, 49.3 ± 6.8, and 48.6 ± 6.2°. Three months later, the FNA on 2D-CT was 49.3 ± 10.5, 42.8 ± 7.4, and 45.1 ± 9.3°, and it was 48.0 ± 6.5, 48.9 ± 7.2, and 49.0 ± 5.7° on 3D-CT, respectively. The FNA in the 3D-PF model at the affected and unaffected hips was 48.5 ± 6.6 and 36.9 ± 13.1°. There were significant differences between 2D-CT and 3D-PF measurements, but no significant difference was found between 3D-CT and 3D-PF measurements. The results by 2D-CT showed significant differences among groups and between the groups. However, the results by 3D-CT had no significant differences among groups or between the groups.

**Conclusion:**

The results of our study showed that 3D-CT is a more precise, and reproducible method for FNA measurement in DDH. The FNA at the affected hips is 11.6° larger than the unaffected in DDH children aged 3–8 years.

## Introduction

Developmental dysplasia of the hip (DDH) is commonly seen in pediatric orthopedics, and pathological characteristics of DDH are shallow acetabulum, enlarged FNA, and deformation of femoral head. In severe cases, the femur can come out of the socket (dislocate) [1]. After abnormal hip joint in children was observed, we can make a preliminary diagnosis by physical examination, and then is confirmed by color ultrasound, X-ray, and CT. Early diagnosis and treatment are very important for the recovery of limb function. For children aged ≤ 18 months old, conservative managements can achieve good outcomes, such as Pavlik harness, cast fixation, and overhead traction. However, especially after 3 years old, most of them needed surgical treatment, such as osteotomy [2]. Femoral neck anteversion angle (FNA) enlargement is also one of the important pathological changes of DDH [3, 4]. In 1954, Billing [5] clearly defined FNA as the angle between the femoral neck axis and condylar plane (determined by the femoral shaft axis and the femoral condyle axis), which has been widely recognized [6]. With technological progress, it is found that FNA is closely related to the hip stress and dysplasia, and is critical for the choice of surgical methods [7, 8]. Therefore, how to accurately measure FNA has always been a hotspot in the orthopedic field.

There are many FNA measuring methods which still remain controversial [3, 9, 10]. Initially, FNA measurement was usually done by biplane X-ray method. However, biplane X-ray method provides spatial superposition information, and especially, the patient position is uncertain while radiographing, so many scholars believe that this method is inaccurate [11, 12]. In recent years, the researchers gradually shifted their focus on how to measure FNA on CT images. In terms of accuracy, reliability, and simplicity, the method of measuring FNA on CT images is better than biplane X-ray method, but some scholars report that the FNA measured on CT by two-dimensional computed tomography (2D-CT) method may still be different from the true value because the femoral neck is an upward and forward three-dimensional structure [13].

Along with the emergence of three-dimensional reconstruction technology and computer aided design, scholars have designed several three-dimensional computed tomography (3D-CT) measurement methods for FNA based on femoral 3D-CT images [14], and such measurement methods have been widely used in clinics. We also have designed a FNA 3D-CT measurement method [15] and found that this method has good stability, repeatability, and high clinical value. However, the accuracy has still raised some doubts [16].

Due to the body particularity of the children with DDH, it is difficult for us to obtain real femur specimens for physical measurement. Therefore, the true value of FNA in children with DDH and whether the FNA measured by our 3D-CT method is close to the true value has not been verified. In recent years, with the rapid development of 3D printing technology, by CT data, we can print a model exactly the same as the human skeleton [17]. 3D printing technology provides ideas for us to verify the accuracy of CT for FNA measurement in children with DDH. In this study, we aimed to measure FNA in children with DDH by 3D-CT and 2D-CT methods, and compare the measured results with the true value obtained by three-dimensional printed femur (3D-PF), and thus evaluate the accuracy of measuring FNA in children with DDH by CT.

### Patients and methods

#### Clinical data

In this retrospective and observational study, 68 patients were enrolled, including 20 males and 48 females, aged from 3 to 8 (5.56 ± 0.81); all the patients had unilateral dislocation of the hip, including 46 patients on the left hip and 22 patients on the right hip. This study was approved by the ethics committee of the Affiliated Central Hospital of Shenyang Medical College (approval no. 20190018) on 15 March 2019 and met the requirements of the Declaration of Helsinki. The legal representative of each patient signed the informed consent.

All patients should meet the following criteria:

Inclusion criteria: (1) suffered from simple hip dislocation, aged 3–8 years old, irrespective of sex; (2) with main clinical manifestations of claudication, pain, or hip instability; (3) diagnosed with DDH by two professors (one radiologist and one orthopedist) with X-ray or CT; (4) received no any treatments before admission.

Exclusion criteria: (1) associated with other congenital malformations; (2) dislocation of the hip caused by inflammation, spasticity, spinal bifida, arthroereisis, or Down’s syndrome.

### Measurement method

The patients were scanned by Philips Brilllance 64-slice spiral CT (scanning conditions: slice thickness 1.5 mm, slice interval 0.5 mm, pitch 0.673, tube voltage 120 kv, tube current 70–120 mA). The CT scanning ranged from anterior superior iliac spine to femoral condyles, and the CT images were sent to the CT workstation (Extended Brilliance Workspace V3.5.0.2250) and the hospital’s picture archive and communication system for measurement. In 2D-CT and 3D-CT measurements, each observer (two radiologists A and B, and one orthopedist C; all were professors) individually completed the measurements, and measured the CT images of patients for the second time after 3 months. However, in 3D-PF measurement, three observers (A, B, and C) jointly and simultaneously measured the FNA of the normal and dislocated femurs.

### 2D-CT measurement method

**Fig. 1:**
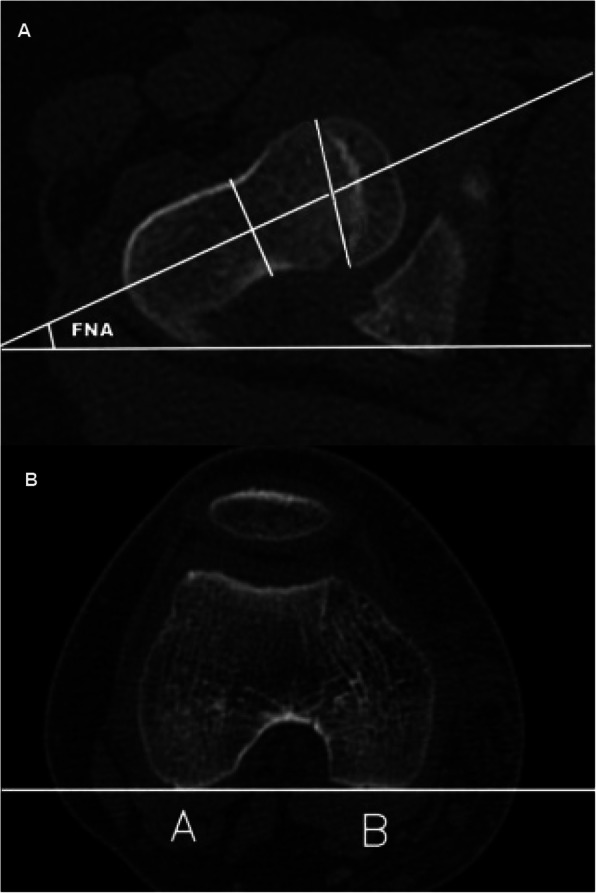
2D-FNA. **a** CT image of the femoral head and the femoral neck. The center of the femoral head and the femoral neck is connected to determine the femoral head-neck axis. **b** It shows the largest layer of the two femoral condyles; the points of A and B are the tangent of the posterior rim of the two condyles. The angle between the femoral head-neck axis and the tangent is 2D-FNA

The most commonly used clinical measurement method described in literature reports was selected [18]. A middle-slice CT image showing both the femoral head and neck was selected, on which the femoral head center and the femoral neck center was then connected to determine the femoral head-neck axis; meanwhile, a CT slice image with the two femoral condyles in the largest size was selected, on which the posterior margins of the medial and lateral femoral condyles were then connected. The angle between the femoral head-neck axis and the line connecting the posterior margins of the medial and lateral femoral condyles was the 2D-FNA (Fig. 1).

### 3D-CT measurement method

**Fig. 2:**
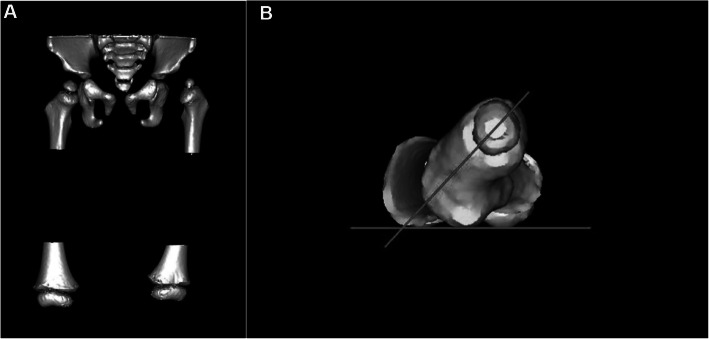
3D-FNA. **a** CT-reconstruction image of the pelvis and the distal and proximal femur. **b** 3D-FNA: the lowest point of the greater trochanter and the medial and lateral femoral condyles are located on a horizontal line by rotating the image. The angle between this horizontal line and the femoral neck axis is the 3D-FNA

After 3D reconstruction of the distal and proximal femoral CT data (Fig. 2a), the image was rotated to overlap the distal and proximal femurs on one plane and have the lowest point of greater trochanter in the middle of the lowest points of medial and lateral femoral condyles, and these three lowest points mentioned above on the same horizontal line. The angle between this horizontal line and the line connecting the femoral head center and the midpoint of the narrowest part of the femoral neck is the 3D-FNA (Fig. 2b).

### 3D-PF measurement method

The DICOM data of the whole length of the femur obtained by CT scanning were extracted and imported into the computer, the femur 3D model was reconstructed by Mimics 10.01 software (Materialise, Leuven, Belgium), and then it was reconstructed with masks. We set the ratio of the reconstructed femur size and the actual femur size as 1:1, saved the data of the reconstructed 3D model of the femur in STL format, and then output the data to quickly print the physical model of the femur 1:1 through a 3D printer (MakerBot, Brooklyn, NY). The material used in the 3D printed femur model is polylactic acid.

**Fig. 3:**
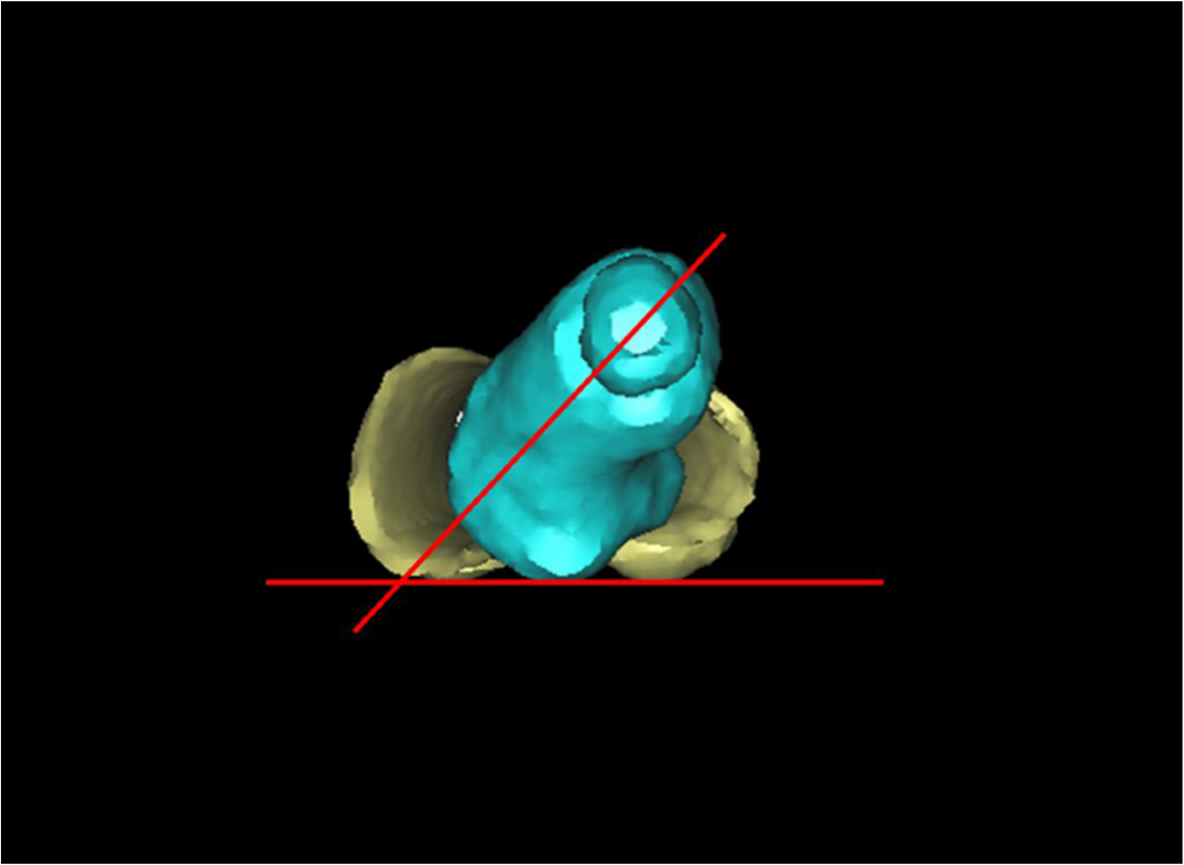
3D PF-FNA. The lowest point of the greater trochanter on the 3D-PF model is located on the same horizontal plane as the medial and lateral condyles of the femur. The center of the femoral head and neck is positioned with a vernier caliper. The angle between these two points and the horizontal plane is measured by electronic angle instrument (called 3DPF-FNA)

The 1:1 physical model of the femur was placed on the horizontal measurement plane, with the medial and lateral femoral condyles and the posterior margin of the greater trochanter in the same plane. The femoral head center and femoral neck center were determined by vernier caliper. The line connecting the femoral head center and the femoral neck center is the femoral neck axis (observing from proximal to distal). An electronic angle measuring instrument was used to measure the angle between the femoral neck axis and the horizontal plane, and such angle is the 3DPF-FNA (Fig. 3).

### Statistical analyses

SPSS software version 23.0 (SPSS company, Chicago, IL) was used for statistical analysis. The consistency of every intra and inter observer between the three groups was analyzed using Pearson correlation coefficient and intra class correlation coefficient (ICC). Based on the bidirectional random effect, absolute consistency, and multi rater measurement model, the ICC estimates of 2D-CT and 3D-CT measurements and the 95% confidence intervals (CI) of inter rater and intra rater reliability were calculated. The interpretation of ICC estimation is as follows: < 0.50, poor; 0.50–0.75, average; 0.75–0.90, good; above 0.90 excellent [19].

Bland-Altman diagram was used to evaluate the consistency among 2D-FNA 3D-FNA and 3DPF-FNA. ANOVA with post hoc tests was used to compare the differences among 2D-FNA, 3D-FNA, and 3DPF-FNA. Independent Student’s *t* test was used to evaluate the difference of FNA angle between dislocated and normal hips. Pearson correlation analysis was used to analyze the correlation between FNAs of dislocated hip and age of children. *P* < 0.05 was considered statistically significant.

## Results

### Results of FNA in 2D-CT, 3D-CT, and 3D-PF measurement

For femurs at the dislocated sides of the 68 patients, in the first measurement, the FNA measured by the three observers were 44.0° ± 6.1°, 49.5° ± 8.9°, and 52.8° ± 7.9° on 2D-CT, respectively, and 47.6° ± 5.4°, 49.3° ± 6.8°, and 48.6° ± 6.2° on 3D-CT. In the second measurement performed 3 months later, the FNA measured by the three observers were respectively 49.3° ± 10.5°°, 42.8° ± 7.4°, and 45.1° ± 9.3° on 2D-CT, and 48.0° ± 6.5°°, 48.9° ± 7.2°, and 49.0° ± 5.7° on 3D-CT. The FNA measured by 3D-PF was 48.5° ± 6.6°. The measurement results of FNA were significantly different compared with 2D-CT and 3D-CT (*P* = .006), and the measurement results of 2D-CT and 3D-PF were also significantly different (*P* = .007). However, there was no significant difference between 3D-CT and 3D-PF (*P* = .081).

**Fig. 4:**
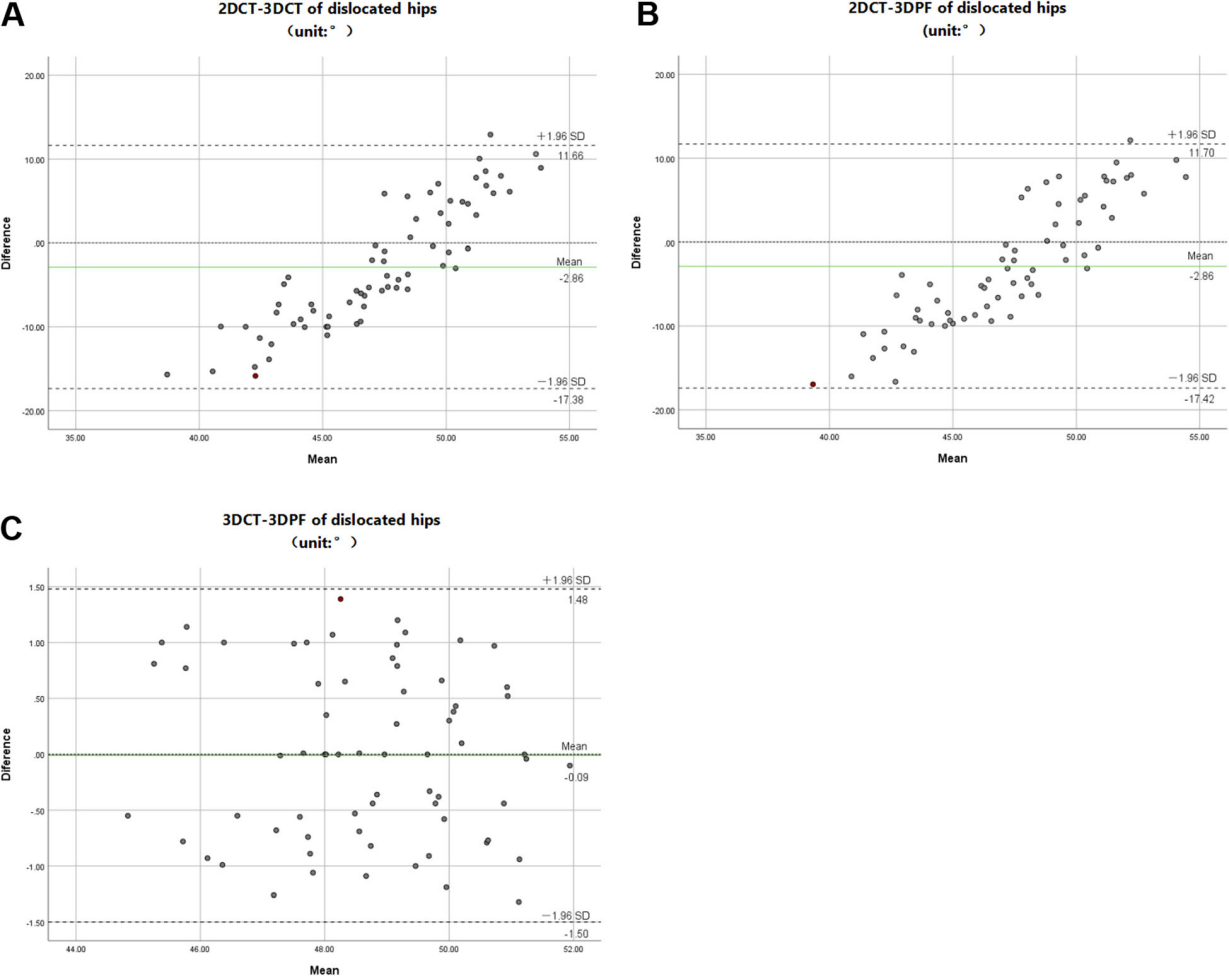
Bland-Altman analysis of different measurements. **a** Compared with the value measured by 2D-CT and 3D-CT, the maximum difference is 15.87 and the mean is 47.06. The results of the two methods are inconsistent. **b** Compared with the value measured by 2D-CT and 3D-PF, the maximum difference is 16.96 and the mean is 47.28. The results of the two methods are inconsistent. Compared with the value measured by 3D-CT and 3D-PF, the maximum difference is 1.39, and the mean is 48.71. The results of the two methods have high consistency

Bland-Altman diagram showed that there was a good consistency in the measurement of FNA in children with DDH between the 3D-CT measurement and 3D-PF measurement. However, there was no consistency in 2D-CT measurements when compared with 3D-CT measurements or 3D-PF measurements (Fig. 4).

The consistency of intra observer and inter observer was poor in the measurement of FNA by 2D-CT. However, FNA’s 3D-CT measurements showed good consistency within the three observers (Table 1). The results showed that the accuracy, stability, and repeatability of 2D-CT method were relatively bad, while the reliability and repeatability of 3D-CT method were satisfied.

**Table 1:** Comparison of intra-observer and inter-observer consistency in the measurement

Observer	2D-FNA		3D-FNA	
	ICC value	95% CI	ICC value	95% CI
A-A	0.452	0.386 to 0.498	0.959	0.931 to 0.986
B-B	0.512	0.465 to 0.563	0.921	0.905 to 0.958
C-C	0.338	0.296 to 0.385	0.856	0.829 to 0.898
A-B	0.442	0.375 to 0.499	0.890	0.875 to 0.921
B-C	0.496	0.466 to 0.513	0.933	0.906 to 0.959
A-C	0.393	0.362 to 0.424	0.968	0.943 to 0.991

A, B, and C represent different observers

### FNA characteristics of the dislocated hips

The FNA of the dislocated hip was larger than that of the normal hip by measuring the real measurement of 3D-PF model. For patients aged 3–8 years with DDH, the FNA of normal hip was 36.9° ± 13.1°, and the FNA of dislocated hip was 48.5° ± 6.6°. Statistical analysis showed that the measurement results of both hip joints were significantly different (*P* = 0.003). The FNA of the dislocated hip increased by approximately 11.6° (mean) compared to the normal hip (Table 2).

**Table 2:** Comparison of FNA in dislocated hip and normal hip groups

	*n*	FNA;°	*P* value
		(mean ± SD)	
Normal hips	68	36.9 ± 13.1	
Dislocated hips	68	48.5 ± 6.6	0.003

The groups were divided based on age; Pearson correlation analysis showed that there was no correlation between age and FNA in the normal hip group (*r* = 0.568; *P* = .093), while in the dislocated hip group, FNA increased with age, and there was a significant positive correlation between age and FNA (*r* = 0.672, *P* = .002) (Table 3).

**Table 3:** Relationship between the FNA and the ages of patients

Age group (years)	*n*	Normal hips (FNA;°)	Dislocated hips (FNA;°)
		(mean ± SD)	(mean ± SD)
3–5	22	35.9 ± 9.1	42.7 ± 9.9
5–7	26	37.8 ± 8.3	48.4 ± 10.7
7–8	20	36.7 ± 9.5	51.6 ± 8.2

Normal hips: *r* = 0.568; *P* = 0.093. Dislocated hips: *r* = 0.672, *P* = 0.002

## Discussion

The treatment for DDH is to achieve concentric reduction of femoral head and acetabulum, so as to restore the normal anatomical structure of hips, which is also the key to evaluate the therapeutic effect after surgeries [20, 21]. Most scholars believe that the FNA increase is one of the important pathological changes of DDH [22]. Proximal femoral derotation osteotomy is often used in surgery for children with DDH to correct the large FNA, so as to achieve concentric reduction of femoral head and acetabulum [23, 24]. However, to determine whether or not a rotation is needed or what the rotation angle is, accurate measurement is required before surgery. The traditional methods mostly depend on the surgeon’s experience to determine the osteotomy rotation angle, which is obviously not accurate and will affect the surgery effect [25]. Therefore, finding an accurate and simple method to measure FNA before surgery is essential.

In the past, many scholars have studied how to accurately measure FNA and invented many FNA measurement methods. However, it is a pity that so far there is no gold standard recognized by most people [26]. At present, CT measurement methods are widely used. There are many different kinds of CT measurement methods [27, 28], where 2D-CT measurement method is the most widely used because of its relatively lower technical requirements, but there is great controversy about the accuracy of the measurement results. A lot of authors think that 2D-CT method is to use 2D images to measure the 3D structure, which is obviously inaccurate, as 2D images cannot simultaneously show the complete structure of the proximal femur [29, 30]. Besides, in 2D-CT measurement method, it is more random and subjective in selection of CT slice images for measurement, which leads to great differences in measurement results between different physicians [31, 32]. In this study, it is found that the difference in results between 3D-PF model and 2D-CT measurement was statistically significant, indicating that the accuracy of 2D-CT measurement method was not very good. In the 2D-CT method for the same 68 patients, the results obtained by the three observers were quite different. In addition, in the 2D-CT measurement for the same patient by the same observer, the difference were still statistically significant in results between the first measurement and the second performed 3 months later, indicating that the stability and repeatability of 2D-CT measurement method in FNA measurement were poor. Therefore, we believe that 2D-CT measurement method is not a reliable method to measure FNA, and applying the data obtained by 2D-CT measurement method to guide clinical surgery, especially proximal femur rotary osteotomy on DDH patients, may lead to severe deviation.

We further analyzed the 2D-CT measurement processes by the three observers, and found that for the same patient, the CT slice images selected by the three observers for measurement were not exactly the same. In addition, in the marking for femoral neck axis and line connecting medial and lateral femoral condyles, the mark points selected are also different between observers. Therefore, we believe that the instability, poor accuracy, and poor repeatability in FNA measurement results of 2D-CT method may be caused by the difference in selection of CT slice images and mark points between the observers.

With 3D-CT technology, we can reconstruct 2D images into 3D images by imaging software. By rotating the 3D images, we can observe the whole from any angle and correct the patient position according to the skeletal coordinates before measurement. The 3D-CT FNA measurement method used in this study is our self-designed method based on the characteristics of the femurs [15]. This method is easy to operate and easy to learn. Since applying this method in the clinic, we have achieved good therapeutic effect, because we calculated the rotation angle of proximal femoral osteotomy based on the FNA measured by 3D-CT method before surgeries, and performed osteotomy according to the calculation result mentioned above. Previous studies have shown that the stability and repeatability in measurement results of our 3D-CT method are significantly better than that of 2D-CT method, but some scholars still question the accuracy of our measurement results [16, 33]. It is difficult to accurately measure the femur structure in human body, and DDH patients are children, so we cannot take the real femur for accurate FNA measurement. Therefore, we have been unable to get the accurate FNA to test the accuracy of 3D-CT measurement method. This has become a problem that bothers us. Although we have achieved good clinical therapeutic effect by applying our 3D-CT measurement method to guide the treatment of DDH patients, there is no data to prove that this method is accurate.

The rapid development of 3D printing technology provides an idea for us to test the accuracy of 3D-CT method in measuring FNA in children with DDH. We collected the CT data of femurs of the 68 children with DDH, and then printed a 1:1 femur model exactly the same as the human skeleton by 3D printing technology, and then performed physical measurement on the model to obtain the accurate FNA of DDH patients. In this study, the FNA in children with DDH measured by 3D-CT method was compared with the real FNA obtained by 3D-PF method, and it was found that there was no statistical difference in the measurement results between 3D-CT method and 3D-PF method, indicating that the FNA in children with DDH measured by 3D-CT method was very accurate. Therefore, it is reliable to use the data obtained by our 3D-CT measurement method to guide femoral osteotomy. By measuring FNA data, we can calculate the increase in FNA to make orthopedics and rotational osteotomy more accurate.

Whether the FNA on the dislocated sides of DDH patients increases is controversial [34]. By measuring FNA of the 68 patients with 3D-PF method, we found that the average FNA of dislocated hips was 48.5 ± 6.6°, and that of normal hips was 36.9 ± 13.1°. There was significant difference between the two results as revealed by statistical test, indicating that the FNA on the dislocated hips in children with DDH generally increases, and the FNA on the dislocated hips in 3–8 years old children with DDH is 11.6° larger than the normal hips. Besides, after assigned by age, we found that after comparing the 3 years old group with the 8 years old group, the FNA of the normal hips had no significant difference. However, the FNA of the dislocated hips enlarged from 42.7 ± 9.9°to 51.6 ± 8.2° significantly. Therefore, it is true that FNA of the dislocated hips in DDH patients is greater than that of the normal hips, and meanwhile, increases with age. In the treatment of DDH patients, the pathological morphology of the increased FNA should be taken into account, and in the surgical treatment of DDH patients, not only acetabulum orthopedics, but also proximal femur rotary orthopedics should be performed.

## Conclusions

3D-CT measurement is a precise, beneficial, and reproducible method for FNA in DDH. Moreover, this method is easy to operate and easy to learn. The FNA at the affected side is 11.6° larger than the healthy side in DDH children aged 3–8 years, and it enlarges with age. In the treatment for children with DDH, we should pay attention to the increase of FNA, and use 3D-CT method to measure FNA to correct FNA timely and accurately. According to the FNA value measured by 3D-CT method, we can accurately correct the rotation angle of the dislocated side in the proximal femoral rotational osteotomy.

Finally, there are some limitations in this study. Firstly, the number of cases in this study is relatively fewer. Secondly, the long-term treatment outcomes of the patients whose FNA measured by 3D-CT have not been followed up and summarized. We will accumulate cases further and complete the full follow-up data to testify the clinical value of 3D-CT method in measuring FNA.

## Data Availability

The data and materials used and/or analyzed during the current study are not publicly available but available from the corresponding author on reasonable request.
